# Deciphering community-level knowledge of obstetric fistula and determinants: perspectives elucidated by ordinal logistic regression

**DOI:** 10.3389/fgwh.2024.1426578

**Published:** 2024-12-23

**Authors:** Miteku Andualem Limenih, Alemakef Wagnew Melesse, Chernet Baye, Beletew Shewabere, Eskedar Getie Mekonnen, Hamid Yimam Hassen, Aster Berhe, Destaye Shiferaw Alemu, Wagaye Fentahun Chanie

**Affiliations:** ^1^ Department of Epidemiology and Biostatistics, Institute of Public Health, College of Medicine and Health Sciences, University of Gondar, Gondar, Ethiopia; ^2^ Department of Obstetrics and Gynecology, School of Medicine, College of Medicine and Health Sciences, University of Gondar, Gondar, Ethiopia; ^3^ Family Health International-360, Addis Ababa, Ethiopia; ^4^ Department of Family Medicine and Population Health, Faculty of Medicine and Health Sciences, University of Antwerp, Antwerp, Belgium; ^5^ VITO Health, Flemish Institute for Technological Research (VITO), Mol, Belgium; ^6^ United Nation Population Fund, Addis Ababa, Ethiopia; ^7^ United Nation Population Fund Supported Maternal Project, University of Gondar, Gondar, Ethiopia

**Keywords:** level of knowledge, obstetric fistula, community level, household, Ethiopia, ordinal regression

## Abstract

**Background:**

Obstetric fistula is a significant cause of maternal morbidity in resource-limited settings, where women often suffer due to a lack of prompt access to skilled obstetric services. It is imperative to comprehend and identify the factors that shape community knowledge about obstetric fistula to enhance prevention strategies, enable early detection, and provide support and treatment to affected women. However, there is a substantial gap in the available evidence concerning the level of community knowledge regarding obstetric fistula and its influencing factors within the Ethiopian context.

**Objective:**

This study aimed to investigate the level of knowledge regarding obstetric fistula and identify influencing factors within the communities in Gondar, Ethiopia.

**Methods:**

A community-based cross-sectional study was conducted among 663 households. Households were selected using a systematic random sampling technique. Descriptive statistics and exact binomial confidence intervals (CIs) were reported. Group comparisons were performed using the Mann–Whitney *U*-test or the Kruskal–Wallis test. Parameters with *p*-value ≤ 0.2 in the univariable ordinal regression were selected for the multivariable ordinal regression model. The proportion odds assumption was checked using the parallel lines test. A multivariable proportional ordinal regression analysis was conducted. *p*-values <5% were considered statistically significant. Data were analyzed using R version 4.3.1.

**Result:**

Of the 663 households, 265 (39.9%; 95% CI: 36.2–43.8), 375 (56.6%; 95% CI: 52.7–60.4), and 23 (3.5%; 95% CI: 2.2–5.2) had low-level, medium-level, and high-level knowledge about obstetric fistula, respectively. Self-employment [adjusted odds ratio (AOR): 0.3; 95% CI: 0.2–0.5], unemployment (AOR: 0.4; 95% CI: 0.2–0.8), income $50–$200 USD (AOR: 5.2; 95% CI: 2–16.1), income > $200 USD (AOR: 7.0; 95% CI: 2.6–22.9), distance from a health facility (AOR: 1.7; 95% CI: 1.1–2.7), non-participation in women’s conferences (AOR: 0.7; 95% CI: 0.5–0.9), and having heard of obstetric fistula (AOR: 4.4; 95% CI: 2.8–7.1) showed statistically significant association with the likelihood of having a high level of knowledge regarding obstetric fistula compared to medium or low levels.

**Conclusion:**

The study reveals an alarmingly low level of knowledge about obstetric fistula. Higher income and prior awareness were associated with increased knowledge about obstetric fistula, while self-employment, unemployment, and non-participation in women's conferences were liked to decreased knowledge. Enhancing community knowledge requires a holistic strategy involving cooperation from all stakeholders and tackling broader socio-economic disparities.

## Introduction

Obstetric fistula is an anomalous conduit linking either the bladder or rectum and the vagina, leading to involuntary leakage of urine or faeces (1, 2). It is one of the most common devastating maternal health problems related to traumatic childbirth injury (3). Globally, an estimated 2.0–3.5 million women are affected by obstetric fistula, with approximately 2 million cases remaining untreated in resource-limited settings (4, 5). Despite being almost entirely preventable and treatable (6), obstetric fistula persists as a significant public and reproductive health concern and serves as a clear indicator of health inequalities and disparities in maternal care worldwide (7, 8).

Each year, millions of women in resource-limited settings endure prolonged labour, leading to the addition of tens of thousands of new fistula cases to the existing millions, causing significant health strain due to complications like faecal and/or urinary incontinence (9). Countries with higher incidences of maternal mortality also have high rates of obstetric fistula for similar reasons (10, 11).

Obstetric fistula remains alarmingly prevalent in regions with limited access to skilled obstetric care, including Ethiopia (12–14). In Ethiopia, more than 110,000 women have suffered from obstetric fistula, resulting in a lifetime risk of experiencing obstetric fistula to be 1,060 per 100,000 women (15). Merely 2% of these women have received treatment within the last 3 years (16), highlighting a significant gap in addressing obstetric fistula. The efforts to eradicate this condition, led by the UN Population Fund and USAID, have been ongoing for over a decade and are essential for achieving the third Sustainable Development Goal (SDG 3) (17, 18). Ethiopia has implemented strategies such as reducing teenage pregnancies, improving access to obstetric care, and establishing treatment modalities to decrease the number of obstetric fistula cases (19, 20). However, the country continues to face 3,300–3,750 new cases of obstetric fistula annually, with a particularly high prevalence in the Amhara region. This is mainly due to a lack of community knowledge that hampers both prevention efforts and timely treatment. In 2016, the Amhara region reported a significant burden of untreated obstetric fistula, with 230 cases per 100,000 women of childbearing age (21). These data underscore the necessity of enhancing community awareness regarding risk factors, prevention methods, and healthcare services to enhance treatment-seeking behaviour and reduce complications.

Competent, prompt, and affordable obstetrical care is essential for preventing obstetric fistula, with community knowledge levels playing a key role in influencing service utilization by overcoming phase 1 delays, as initial delays in seeking care often stem from a lack of recognizing prolonged labour and limited awareness of the benefits of timely care and treatment, underscoring the importance of understanding the causes, prevention, and treatment of obstetric fistula to reduce its impact significantly (8, 9, 17, 22–24). Therefore, good knowledge of obstetric fistula is pivotal in addressing its burden, and extending care beyond surgery to include community reintegration is crucial for the holistic wellbeing of affected women (25, 26).

Understanding the community knowledge level of obstetric fistula and its determinant factors is paramount for developing effective interventions and reducing its prevalence. While numerous studies have explored obstetric fistula from a clinical and epidemiological perspective (4, 15, 19, 22, 27, 28), few have delved deeply into the community’s understanding of this condition and its underlying causes (15, 21, 27).

Community's knowledge of obstetric fistula and its associated factors remains a significant challenge in Ethiopia, especially in the Amhara region, where the burden is unacceptably high. Previous studies conducted in health centres among fistula victims, who likely have higher knowledge levels than the background population, cannot be extrapolated to assess community knowledge, and despite the crucial role that partners play in disease stigmatization, existing research fails to evaluate community knowledge about obstetric fistula, including the perspectives of men.

Increasing the level of knowledge about obstetric fistula in the community and tackling its underlying factors are vitally important to help victims receive the required social support and, in turn, reduce the increasing number of fistula sufferers (6, 22, 25, 29). However, there is a substantial gap in the available evidence concerning the level of community knowledge regarding obstetric fistula and its influencing factors, particularly in the study area. This study sought to bridge this gap by assessing the collective knowledge about obstetric fistula among community members, including men. Therefore, this study aimed to assess the community knowledge level about obstetric fistula and its associated factors in Gondar, Ethiopia.

## Materials and methods

### Study design, period, and setting

A community-based cross-sectional study was conducted from 1 January 2024 to 20 February 2024. The study was conducted in Gondar City Administration in the Amhara region of Ethiopia. The city constitutes 12 administrative areas or sub-cities consisting of 22 kebeles (the smallest administrative unit in Ethiopia), hosting approximately 53,725 households and 395,000 adults. The city has one referral hospital with a fistula treatment centre and eight governmental health centres.

### Source and study population

The source population for this study included both male and female adults aged 18 years and above. The study population consisted of individuals aged 18 years and above who had been living in the city administration for at least 6 months. Exclusion criteria included unwillingness to participate in the study and living in the city for less than 6 months.

### Sample size determination and sampling technique

The single population proportion formula was used to calculate the sample size for the study, considering a 95% confidence level (with a corresponding *Z*-score of 1.96), a 50% proportion of the population (P), and a 4% margin of error (W), which yielded 601 participants. Taking into account a 10% non-response rate, a total of 663 adults were included in this study.

From the 22 kebeles in the city, 5 kebeles (20% of the total kebeles) were selected randomly using the lottery method to ensure representativeness, and samples were proportionally allocated based on the number of households in each selected kebeles. Then, households were selected using systematic random sampling with a sampling fraction of 14 (*k* = *n_i_*/*N_i_*, where *n_i_* is the proportionally allocated sample from each selected kebeles and *N_i_* represents the total number of households from the respective kebeles). The first household was selected using simple random sampling (from 1 to 14), and then every 14th household was included in the study. Finally, one person aged 18 years or above was selected from each household as a study participant using the lottery method.

### Study variables

The dependent variable for this study was the level of knowledge about obstetric fistula, categorized into three levels: low, medium, and high. The independent variables included socio-demographic characteristics including age, gender, religion, marital status, educational status, occupation, monthly income, family size, distance from a health facility, and obstetric-related medical and general conditions.

### Measurement of the outcome variable

Adults’ knowledge about obstetric fistula was assessed using 10 questions comprising 32 items, which mainly evaluated whether participants had heard of obstetric fistula, knowledge of its risk factors, knowledge of preventive measures, understanding of types of obstetric fistula, awareness of the causes of obstetric fistula, ability to recognize obstetric danger signs, identification of signs and symptoms of obstetric fistula, knowledge of complications, and awareness of treatment options for obstetric fistula. The response for each item was scored as one point if correct and zero otherwise. Therefore, the possible score for a respondent ranged from 0 to 32. The overall knowledge score for an individual was calculated by summing all correct responses, and the overall score for each respondent was categorized into three levels of knowledge regarding obstetric fistula based on Bloom's cut-off: high, medium, and low (30). Study participants whose correct score ranges from 80% to 100% (≥26 correct responses) were classified as having a high level of knowledge, those scoring from 60% to 79% (≥20 and <26 correct responses) were classified as having a medium level of knowledge, and those with a score of <60% (<20 correct responses) were classified as having a low level of knowledge about obstetric fistula. In addition to the outcome variable, we defined “active participation in women's conferences” as attending at least one of the two women's conferences held each month, whether by women or men.

### Data collection methods and procedures

Before going to the field for data collection, a 1-day training was provided to data collectors about the purpose of the study, data collection tools and techniques, and ethical issues during the participant selection and data collection. Data were collected using a face-to-face, interviewer-administrated, pretested, semi-structured questionnaire, which was adapted through a review of relevant literature. The internal consistency of the questionnaire was estimated using Cronbach’s alpha, and was found to be good (>0.70). The data were collected via an open-source Kobo Toolbox, which took approximately 50–60 min. The data were subsequently exported to an Excel spreadsheet (version 2108, Microsoft Corp) for further processing and analysis.

### Statistical analysis

Descriptive statistics were reported using absolute and relative frequencies; for normally distributed data, means with standard deviations were presented, whereas medians with interquartile ranges (IQRs) were used for non-normally distributed data. Exact binomial confidence intervals (CIs) were reported for relative frequencies unless stated otherwise. Group comparisons were performed using the Mann–Whitney *U*-test or the Kruskal–Wallis test. To account for the ordinal nature of the outcome, various ordinal logistic regression model exists. Particularly, in line with the interpretability of our research findings, the proportional odds model (POM) was deemed more rational and understandable. This model is also frequently utilized in epidemiological and biological applications. Hence, the proportional odds model was used. The likelihood ratio test and Akaike information criterion (AIC) were used to evaluate the choice between the proportional odds model and the partial proportional odds model (PPOM). Univariable ordinal regression was performed to get insights into the determinants of the level of knowledge about obstetric fistula. Variables with a *p*-value ≤0.2 in the univariable ordinal regression were fitted into the multivariable ordinal regression model. The proportional odds assumption was evaluated through the parallel lines test. A multivariable proportional ordinal regression model was built using the level of knowledge categories and all potential covariates. The results were presented as adjusted odds ratios with corresponding 95% CIs. A two-sided test was used for all hypotheses, with a significance level set at 5% (*p* < 0.05). Data were analyzed using the free statistical software R, version 4.3.1 (R Project for Statistical Computing).

### Ethical consideration

Ethical approval was obtained from the University of Gondar (VP/S/10291). An official permission letter was obtained from the Gondar City Administration. Support letters were obtained from the selected kebele administrative offices. Verbal informed consent was obtained from household heads and individual study participants after the purpose of the study was explained to them. Confidentiality was ensured by avoiding identifiers. Each participant was informed that participation in the study was entirely voluntary. Participants who are potentially at high risk of obstetric fistula were provided with additional information to encourage them to evaluate themselves and receive service at the University of Gondar referral hospital for detailed examination and appropriate management.

## Results

### Socio-demographic characteristics of the respondents

Six hundred sixty-three adults were involved in this study, with a median age of 32.0 years (IQR: 19.0–64.0). Slightly more than half (50.7%) of the study participants were women, and 46.2% (*n* = 306) of the respondents had secondary education as their highest level of education. Of the 663 participants, 456 (68.8%) were married, and about 2 in 5 (42.8%) were employed by the government. The average monthly household monthly income was 8,010 ETB (Table 1).

**Table 1:** Socio-demographic characteristics of respondents, Gondar Town, Ethiopia, 2024.

Characteristics	Number (*n*)	Percentage
Age (years)		
<20	27	4.1
20–30	255	38.5
31–40	237	35.7
41–49	85	12.8
>49	59	8.9
Gender		
Female	336	50.7
Male	327	49.3
Religion		
Catholic	6	0.9
Protestant	15	2.3
Muslim	45	6.8
Orthodox	597	90.0
Marital status		
Widowed	13	2.0
Divorced	22	3.3
Single	172	25.9
Married	456	68.8
Educational status		
No formal education	52	7.8
Primary education	189	28.5
Secondary education	306	46.2
College and above	116	17.5
Occupation		
Unemployed	44	6.6
Self-employed	157	23.7
Private employee	178	26.8
Government employee	284	42.8
Monthly income (ETB)		
<2,250	49	7.4
2,250–9,000	373	56.3
>9,000	241	36.3
Family size		
1–2	47	7.1
3–5	408	61.5
≥6	208	31.4
Distance from the health facility (min)		
>30	206	31.1
≤30	457	68.9

### Obstetric fistula-related characteristics of the study participants

In this study, a substantial majority of the participants, 567 (85.5%), did not receive counselling on obstetric fistula, including information regarding its risk factors, preventive measures, and management options. In addition, only 280 study participants (42.2%) had heard of obstetric fistula through various sources, including social media, print media, healthcare professionals, family members, or friends. Furthermore, healthcare providers informed only a small fraction of individuals (1.1%) about having signs of obstetric fistula (Table 2).

**Table 2:** Obstetric fistula-related and medical characteristics of the respondents at Gondar, 2024.

Variables	Number (*n*)	Percentage
Aware of obstetric danger signs		
Yes	300	45.2
No	363	54.8
Ever counselled about obstetric fistula		
Yes	96	14.5
No	567	85.5
Ever participated in women’s conferences		
Yes	203	30.6
No	460	69.4
Have any chronic disease		
Yes	123	18.6
No	540	81.4
Perception of experiencing health issues related to obstetric fistula (signs, symptoms, or complications)		
Yes	91	13.7
No	572	86.3
Ever heard of obstetric fistula		
Yes	280	42.2
No	383	57.8
Ever experienced leakage of urine		
Yes	94	14.2
No	569	85.8
Healthcare visit because of perceived obstetric fistula symptoms		
Yes	58	8.7
No	605	91.3
Ever been told of having obstetric fistula signs by a healthcare provider		
Yes	7	1.1
No	656	98.9

### Medical profiles of study participants

Of the total participants, 123 individuals (18.6%) reported experiencing at least one chronic disease, where more than a third (35.8%) reported diabetes mellitus, and among all adults with chronic diseases, 71 (57.7%) and experienced hypertensive disorders and 8 (6.5%) reported other chronic comorbidities.

### Knowledge of the study participants about obstetric fistula

Three hundred ninety-eight respondents, accounting for 60.0% of the total, demonstrated a good understanding of the signs, symptoms, risk factors, complications, and prevention methods of obstetric fistula (Table 3).

**Table 3:** Comprehensive knowledge of study participants about obstetric fistula at Gondar, 2024.

Variables	Category	Frequency (*n*)	Percentage
What is obstetric fistula	Communication between the birth canal and bladder or rectum	181	27.3
	Urinary or faecal incontinence/leakage	106	16.0
	Health problems for women	105	15.8
	Infection of the reproductive tract	100	15.1
	I do not know	171	25.8
Signs and symptoms of obstetric fistula	Urinary incontinency	446	67.3
	Faecal incontinency	307	46.3
	Vulval irritation	496	74.8
	Foul-smelling vaginal discharge	321	48.4
	Leakage of gas/faeces into the vagina	263	39.7
	Pain while having sex	353	53.2
	I do not know	546	82.4
Myths and conceptions about obstetric fistula	Non-violent sex or promiscuity causes fistula	513	77.4
	Holding urine for an extended time causes fistula	515	77.7
	Existing medical condition causes fistula	551	83.1
	Digital PV examination causes fistula	521	78.6
	Fistula relates to multiple pregnancies	164	24.7
	Poor personal hygiene causes fistula	479	72.2
Direct causes of fistula	Prolonged/obstructed labour causes fistula	536	80.8
	Provider error in C/S and abortion causes fistula	316	47.7
	Traumatic sexual violence causes fistula	510	76.9
	Big baby birth causes fistula	318	48.0
	Improper forceps delivery causes fistula	107	16.1
Indirect causes of fistula	Early marriage can cause obstetric fistula	548	82.7
	Teenage pregnancy can cause obstetric fistula	67	10.1
	Mismanagement of delivery by TBA can cause fistula	159	24.0
	Delays in healthcare (ANC and labour) can cause fistula	315	47.5
Concepts on prevention of obstetric fistula	Obstetric fistula is preventable	573	86.4
	Obstetric fistula is a treatable condition	91	13.7
	Know when fistula treatment is available	161	24.3
Prevention methods of obstetric fistula	Delaying the age of the first pregnancy	534	80.5
	Cessation of harmful traditional practice	307	46.3
	Timely seeking skilled maternal healthcare	421	63.5
	Skilled care at birth/institutional delivery	271	40.9
	Using family planning methods	326	49.2

PV, per vaginal examination; C/S, cesarean section; TBA, traditional birth attendant, ANC, antenatal care.

**Figure 1:**
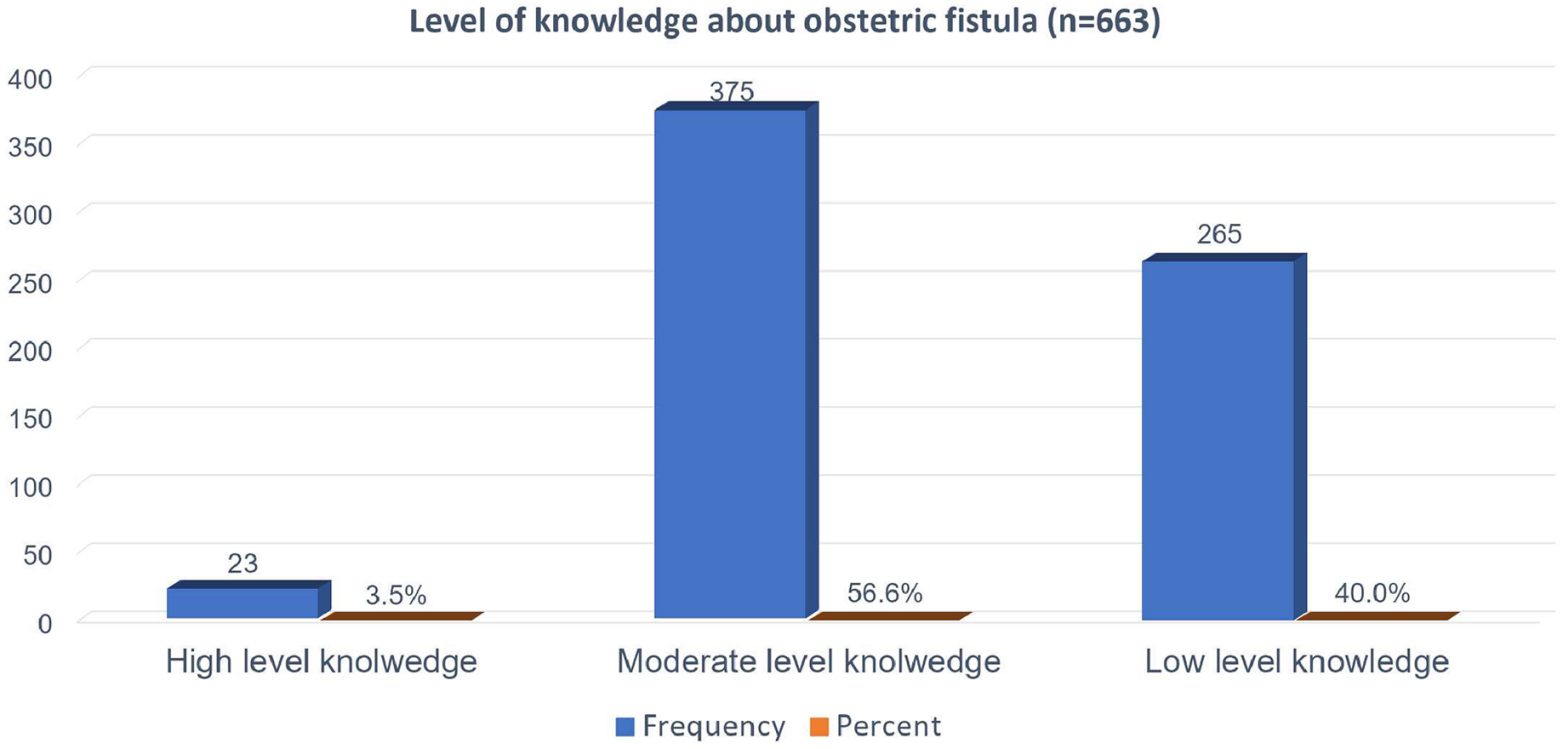
Individual’s level of knowledge about obstetric fistula among study participants in Gondar, 2024.

Furthermore, from the total study participants, an unacceptably high number of individuals, comprising 265 (40.0%), were categorized as having a low level of knowledge about obstetric fistula (Figure 1).

### Univariable ordinal logistic regression analysis

Several variables were found to have a statistically significant association with the level of knowledge about obstetric fistulas. These variables include age of the respondent, marital status, education level, occupation, average monthly income, family size, distance from the nearest health facility, participation in women's health conferences, awareness of obstetric danger signs, perception of experiencing obstetric fistulas, having healthcare visits related to perceived obstetric fistula symptoms, experience with urine leakage, and having ever heard of obstetric fistulas (Table 4).

**Table 4:** Summary of univariable ordinal regression analysis by the level of knowledge about obstetric fistula.

Parameters	Level of knowledge (*N* = 663)			
	Low *N* (%; 95% CI)	Medium *N* (%; 95% CI)	High *N* (%; 95% CI)	*p*-value
Observations, *N*	265 (39.9; 36.2–43.8)	375 (56.6; 52.7–60.4)	23 (3.5; 2.2–5.2)	
Age, median (IQR)	29 (19–64)	35 (19–63)	33 (20–45)	<0.001
Educational status				
No formal education	26 (9.8; 6.5–14.0)	25 (6.7; 4.3–9.7)	1 (4.3;0.1–21.9)	0.069
Elementary education	87 (32.8;27.2–38.8)	93 (24.8; 20.5–29.5)	9 (39.1;19.7–61.5)	
Secondary education	106 (40.0; 34.1–46.2)	191 (50.9; 45.8–56.1)	9 (39.1;19.7–61.5)	
College and above	46 (17.4; 12.9–22.5)	66 (17.6; 13.9–21.8)	4 (17.4; 4.9–38.8)	
Marital status				
Widowed	3 (1.1; 0.2–3.3)	10 (2.7; 1.2–4.8)	0 (0.0; 0.0–14.8)	<0.001
Divorced	8 (3.0; 1.3–5.9)	14 (3.7; 2.1–6.2)	0 (0.0; 0.0–14.8)	
Single	96 (36.2; 30.4–42.3)	69 (18.4; 14.6–22.7)	79 (30.4; 13.2–52.9)	
Married	158 (59.6; 53.4–65.6)	282 (75.2; 70.5–79.5)	16 (69.6; 47.1–86.8)	
Occupation				
Unemployed	24 (9.1; 5.9–13.2)	19 (5.1; 3.1–7.8)	1 (4.3; 0.1–21.9)	<0.001
Self-employed	97 (36.6; 30.8–42.7)	60 (16.0; 12.4–20.1)	0 (0.0; 0.0–14.8)	
Private employee	59 (22.3; 17.4–27.8)	109 (29.1; 24.5–33.9)	10 (43.5: 23.2–65.5)	
Government employee	85 (32.1; 26.5–38.1)	187 (49.9; 44.7–55.0)	12 (52.2; 30.6–73.2)	
Average monthly income (ETB)				
<2,250	44 (16.6; 12.3–21.6)	5 (1.3; 0.4–3.1)	0 (0.0; 0.0–14.8)	<0.001
2,250–9,000	159 (60.0; 53.8–65.9)	197 (52.5; 47.3–57.7)	17 (73.9; 51.6–89.8)	
>9,000	62 (23.4; 18.4–28.9)	173 (46.1; 41.0–51.3)	6 (26.1; 10.2–48.4)	
Family size				
1–2	35 (13.2; 9.4–17.9)	12 (3.2; 1.6–5.5)	0 (0.0; 0.0–14.8)	<0.001
3–5	175 (66.0; 59.9–71.7)	214 (57.1; 51.9–62.1)	19 (82.6; 61.2–95.0)	
≥6	55 (20.8; 16.0–26.1)	149 (39.7; 34.7–44.9)	4 (17.4; 4.9–38.8)	
Distance from a health facility (min)				
>30	132 (49.8; 43.6–55.9)	71 (18.9; 15.1–23.3)	3 (13.0; 2.8–33.6)	<0.001
≤30	133 (50.2; 44.0–56.4)	304 (81.1; 76.7–84.9)	20 (87; 66.4–97.2)	
Ever participated in women’s conferences				
No	170 (64.2; 58.1–69.9)	269 (71.7; 66.9–76.2)	21 (91.3; 71.9–98.9)	0.005
Yes	95 (35.8; 30.1–41.9)	106 (28.3; 23.7–33.1)	2 (8.7; 1.1–28.0)	
Aware of obstetric danger signs				
No	158 (59.6; 53.4–65.6)	190 (50.7; 45.5–55.8)	15 (65.2; 42.7–83.6)	0.087
Yes	107 (40.4; 34.4–46.6)	185 (49.3; 44.2–54.5)	8 (34.8; 16.4–57.3)	
Perception of experiencing health issues related to obstetric fistula				
No	247 (93.2; 89.5–95.9)	306 (81.6; 77.3–85.4)	19 (82.6; 61.2–95.0)	<0.001
Yes	18 (6.8; 4.1–10.5)	69 (18.4; 14.6–22.7)	4 (17.4; 4.9–38.8)	
Healthcare visit because of perceived obstetric fistula symptoms				
No	251 (94.7; 91.3–97.1)	331 (88.3; 84.6–91.3)	23 (100.0; 85.2–100)	0.036
Yes	14 (5.3; 2.9–8.7)	44 (11.7; 8.6–15.4)	0 (0.0; 0.0–14.8)	
Ever experienced leakage of urine				
No	243 (91.7; 87.7–94.7)	309 (82.4; 78.1–86.1)	17 (73.9; 51.6–89.8)	<0.001
Yes	22 (8.3; 5.3–12.3)	66 (17.6; 13.9–21.8)	6 (26.1; 10.2–48.4)	
Ever heard of obstetric fistula				
No	220 (83.0; 77.9–87.3)	157 (41.9; 36.8047.0)	6 (26.1; 10.2–48.4)	<0.001
Yes	45 (17.0; 12.7–22.10)	218 (58.1; 52.9–63.2)	17 (73.9; 51.6–89.8)	

### Multivariable ordinal logistic regression analysis

The multivariable proportional ordinal regression analysis, following covariate adjustment, identified that occupation, average monthly income, distance from a health facility, participation in women’s conferences, and awareness (heard) of obstetric fistula were all statistically significant independent factors influencing the level of knowledge about obstetric fistula.

The odds of demonstrating a high level of knowledge about obstetric fistula, compared to a medium or low level, were reduced by 67.3% (adjusted odds ratio: 0.327; 95% CI: 0.196–0.541) for self-employed individuals compared to those employed by the government. Similarly, unemployed individuals had 0.352 times lower odds (95% CI: 0.154–0.793) of attaining high-level knowledge than government-employed individuals.

The odds of having a high level of knowledge about obstetric fistula, compared to a medium or low level, were 5.161 times greater (95% CI: 2.012–16.107) for individuals with an average monthly income between 2,250 and 9,000 ETB and 7.013 times higher (95% CI: 2.561–22.907) for those with an income exceeding 9,000 ETB, compared to individuals earning less than 2,250 ETB.

The likelihood of having a high level of knowledge about obstetric fistula, compared to a medium or low level, was 1.7 times higher (95% CI: 1.006–2.699) for individuals residing within a distance from a health facility requiring ≤30 min of travel time, compared to those residing in regions where travel time exceeds 30 min.

The likelihood of having a high-level knowledge about obstetric fistula, compared to a medium or low level, was 33.8% lower [adjusted odds ratio (AOR): 0.662; 95% CI: 0.445–0.983] for individuals who did not actively participate in women's conferences compared to those who did. Furthermore, among individuals within the household who received information about obstetric fistula through various media channels, the odds of achieving a high level of knowledge, relative to a medium or low level, were 4.443 times higher (95% CI: 2.821–7.094) than for those who did not receive such information (Table 5).

**Table 5:** Multivariable proportional ordinal regression using the level of knowledge as a response.

Parameters	Estimate	SE	Adjusted OR (95% CI)	*p*-value
Occupation				
Government employee	1	1	1	1
Self-employed	−1.118	0.258	0.327 (0.196–0.541)	<0.001
Unemployed	−1.043	0.416	0.352 (0.154–0.793)	0.012
Monthly income (ETB)				
<2,250	1	1	1	1
2,250–9,000	1.641	0.521	5.161 (2.012–16.107)	0.001
>9,000	1.948	0.549	7.013 (2.561–22.907)	<0.001
Distance from a health facility (min)				
>30	1	1	1	1
≤30	0.497	0.251	1.645 (1.006–2.699)	0.047
Participated in women’s conferences				
Yes	1	1	1	1
No	−0.412	0.201	0.662 (0.445–0.983)	0.041
Aware (heard) of obstetric fistula				
No	1	1	1	1
Yes	1.491	0.235	4.443 (2.821–7.094)	<0.001

## Discussion

The results of this multivariable proportional ordinal regression analysis indicate that an unacceptably high number of study participants, two out of five (39.9%), had a low level of knowledge about obstetric fistula.

The results of our study appear to align closely with the findings from research conducted in Ghana, which reported a similar low level of knowledge at 37.2% (31). This similarity could be attributed to the fact that both studies were carried out in urban settings, potentially leading to comparable demographic compositions. However, the findings of our research are notably lower than the statistics reported in the 2016 Ethiopian Demographic Health Survey (61.0%) (15), as well as the study reports from Bench Sheko Zone at 59.2% (19) and northwest Ethiopia (63.6%) (32). This variance may be attributed to differences in the study population, as their research encompassed both urban and rural demographics and included solely women. In addition, the disparity could be linked to variations in the study period. Recent substantial efforts have been made to implement health campaigns aimed at raising awareness, prevent obstetric fistula, and provide referral and linkage programs for obstetric fistula. These efforts likely contributed to an enhanced understanding of obstetric fistula among households in our study (9, 25, 26). Furthermore, the consistent utilization of communication media over time has played a significant role. Media platforms possess a potent capacity for disseminating information, thereby potentially elevating the knowledge levels of individuals within households (22, 25, 29, 33).

The odds of demonstrating a high level of knowledge about obstetric fistula, compared to a medium or low level, was reduced by 67.3% for self-employed individuals compared to those employed by the government. Similarly, unemployed individuals exhibited 0.352 times lower odds than government-employed individuals in attaining high-level knowledge vs. medium or low levels. This result is substantiated by the findings reported by Dejen et al. (32), Azanu et al. (24), and Wisdom et al. (31). This might be because the lower levels of knowledge about obstetric fistula among the self-employed and unemployed individuals may be attributed to limited access to formal healthcare education and information due to potential financial constraints and lack of consistent exposure to healthcare resources.

Adults with a relatively higher monthly household income demonstrated a higher level of fistula-related knowledge, and it is clear that there is a gradient of knowledge across the income categories. Individuals who secured a monthly household income of 9,000 and above ETB were more privileged to know more about fistula than those with lower household income categories. Taking income as a proxy measure for socio-economic status (34), this suggests that socio-economic status plays a vital role in knowledge about obstetric fistula. Individuals with higher income might have better access to healthcare education resources, which could contribute to their understanding of issues related to obstetric fistula. Supporting evidence was reported from community-based studies in Ethiopia, where women with media exposure, such as television or radio, had a better understanding of the health issues (27, 28). Media serve as an essential tool for obtaining up-to-date available information that enhances public knowledge (35). This underscores the importance of providing targeted awareness-raising activities about fistula to community members with lower income to bridge the existing knowledge gap. Furthermore, the findings highlight the need for specifically oriented programs to reach lower-income groups through educational workshops and informational materials tailored to the needs of this segment of the community.

Distance to the nearby health facility significantly influences the obstetric fistula-related level of knowledge among adult community members. Individuals who need to commute for less than or up to 30 min to access healthcare facilities were more likely to possess adequate knowledge about obstetric fistula; conversely, those who travel for more than 30 min were less likely to have such knowledge. This finding suggests a correlation between the distance individuals travel to access healthcare and their knowledge. Such reports were observed from studies in Ethiopia (36) and China (37). Individuals with shorter commutes to healthcare facilities may belong to communities with better access to education, be motivated to seek healthcare, interact frequently with healthcare providers, or have greater exposure to fistula health education initiatives. Further research is needed to explore the observed association for in-depth understanding.

Lack of participation in discussions concerning women's affairs was a significant factor that affected the level of obstetric fistula-related knowledge among adults. Individuals who did not actively participate in conferences on issues related to women were less likely to possess essential knowledge about obstetric fistula, which highlights the importance of active involvement of adult community members in dealings that focus on issues related to women.

Awareness of obstetric fistula has a significant contribution to fistula-related level of knowledge among adults. Individuals who have heard of obstetric fistula were over 4.44 times more likely to be knowledgeable compared to those who have not, indicating that knowledge disparities may stem from variations in socio-demographic, economic, and community-related factors within the community (27). However, despite this increased awareness, significant misconceptions about the causes of obstetric fistula persist within the community. A notable proportion of respondents incorrectly attribute the condition to non-violent sex or promiscuity (77.4%) and holding urine for extended periods (77.7%). These misconceptions align with findings from studies by Tsega Dejen et al. and Animut et al., who reported similar misunderstandings (4, 28). In addition, factors such as poor personal hygiene (72.2%), digital pelvic examinations (78.6%), and existing medical conditions (83.1%) are also mistakenly believed to cause fistula, as observed in the study by Bulndi et al. (38). These widespread misconceptions highlight a critical gap in community knowledge that could hinder efforts to prevent and treat obstetric fistula. Therefore, addressing these myths through targeted education programs is essential for promoting an accurate understanding of the true causes, which are primarily linked to prolonged obstructed labour.

### Strengths and limitations

One notable aspect of this study is the inclusion of male participants, often overlooked in previous studies. It is important to recognize that men also play a role in addressing this issue, although obstetric fistula affects women. Furthermore, unlike most previous studies on the same topic, this study provides a comprehensive understanding of fistula knowledge within the general population, which is vitally important for developing holistic approaches that can effectively facilitate interventions to curb the devastating impacts of fistula. However, despite these strengths, the findings from this study should be interpreted in light of the following limitations. First, the study was conducted in a town with relatively educated people, and it excludes rural residents, who may have a different understanding of the condition. These may overestimate the proportion of knowledge.

### Recommendations

The study reveals an alarmingly low level of knowledge about obstetric fistula. Higher income and prior awareness increased the level of knowledge about obstetric fistula, while self-employment, unemployment, and non-participation in women's conferences decreased it. Addressing broader socio-economic disparities is essential for improving knowledge about obstetric fistula among adults in the study area. Initiatives to reduce income inequality, improve access to education, and provide social support for marginalized communities may be considered. Policymakers should consider socio-economic factors when developing health policies related to obstetric fistula with other maternal health concerns. Further studies in other settings, including people in the countryside, should be considered to understand the overall picture. It is also recommended to assess how the level of knowledge leads to action to prevent and minimize obstetric-related impacts in the community.

## Data Availability

The original contributions presented in the study are included in the article/Supplementary Material, further inquiries can be directed to the corresponding author.
